# GPS Multipath Analysis Using Fresnel Zones

**DOI:** 10.3390/s19010025

**Published:** 2018-12-21

**Authors:** Florian Zimmermann, Berit Schmitz, Lasse Klingbeil, Heiner Kuhlmann

**Affiliations:** Institute of Geodesy and Geoinformation, University of Bonn, D-53115 Bonn, Germany; s7beschm@uni-bonn.de (B.S.); klingbeil@igg.uni-bonn.de (L.K.); kuhlmann@uni-bonn.de (H.K.)

**Keywords:** GPS, Fresnel zones, multipath, signal diffraction, obstruction adaptive elevation masks

## Abstract

GNSS (Global Navigation Satellite Systems) multipath has been subject to scientific research for decades and although numerous methods and techniques have already been developed to mitigate this effect, it is still one of the accuracy-limiting factors in many GNSS applications. Since multipath is highly dependent on the individual antenna environment, there is still a need for new methods and further investigations to increase the understanding of this systematic effect. In this paper, the concept of Fresnel zones is applied to two different aspects of multipath. First, Fresnel zones are determined for the line-of-sight transmission between satellite and receiver. By comparing the boundary of the Fresnel zones to an obstruction adaptive elevation mask, potentially diffracted signals can be identified and excluded from the position estimation process. Both the percentage of epochs with fixed ambiguities and the positioning accuracy can be increased by the proposed method. Second, Fresnel zones are used to analyze the multipath induced by a horizontal and spatially-limited reflector. The comparison of simulated and real signal-to-noise (SNR) observations reveals a relationship between the percentage of the overlap of the Fresnel zone and reflector and the occurrence of multipath. It is found that an overlap of 50% is sufficient to induce multipath effects. This is of special interest, since this does not confirm theoretical assumptions of the multipath theory.

## 1. Motivation

The signals from GNSS (Global Navigation Satellite Systems) satellites can be used to estimate the position of the user antenna. Especially in applications with high accuracy requirements, appropriate countermeasures have to be applied to account for the systematic observation errors, influencing the accuracy of the positioning solution. In differential approaches, the position of the user antenna is determined relative to a master antenna. By forming double-differences of the observations simultaneously received at the master and user antenna, the majority of systematic errors, such as orbital and atmospheric errors, can be eliminated or at least minimized. After this processing step, site-dependent effects remain accuracy-limiting factors, since these errors depend on the individual antenna environment and cannot be eliminated by differential techniques.

Although site-dependent errors are often generalized as multipath, it is necessary to distinguish between four effects: (1) far-field multipath, (2) non-line-of-sight (NLOS) reception, (3) signal diffraction and (4) near-field effects. Far-field multipath occurs when the direct GNSS signal that has arrived through the line-of-sight (LOS) path is superimposed by a reflected signal that reaches the antenna on one or more indirect paths [1]. If the direct signal is completely blocked by an obstacle and only the reflected signal arrives at the antenna, one speaks of NLOS reception [2]. Signal diffraction describes the phenomena where the direct signal path is also blocked but the signal is diffracted, for example, at an edge of an obstacle [3]. Finally, near-field effects arise from the closest vicinity of the antenna and can lead to an altering of the electrodynamic properties of the antenna itself [4]. However, in this paper, we focus on far-field multipath effects and signal diffraction, and for the sake of simplicity, far-field multipath is referred to as multipath hereafter.

Besides the distinction between the aforementioned effects, it is also necessary to discriminate between code- and carrier-phase multipath. Depending on the severity of multipath conditions, the code-multipath error can range between several meters up to 100 m, while the carrier-phase multipath error is limited to a few centimeters [1]. Thus, in the case where both observation types are used, the code-multipath error will be the dominant influence. Especially for applications where an instantaneous carrier-phase ambiguity resolution is needed, this is a crucial factor. Since the dimensions of the search space for the ambiguity resolution usually depend on the accuracy of the code observations, the code-multipath can enlarge the search space, and thus, the ambiguity resolution can take longer [5]. As a consequence, positioning techniques that only use carrier-phase observations, e.g., as presented in [6], are of special interest, since such approaches eliminate the dominant influence of the code-multipath.

The majority of multipath mitigation techniques are either data-driven approaches, or they are related to the receiver architecture or special antenna design. A detailed overview of multipath mitigation techniques can be found in [7].

Data-driven approaches, such as sidereal filtering [8,9,10,11], multipath stacking maps [12,13,14] or signal-to-noise (SNR)-based filtering and modeling [3,15,16,17], have been developed to minimize the influence of multipath interference. The purpose of receiver-internal techniques, such as different correlator spacings or the Multipath Estimation Delay-Lock-Loop [18], and special antenna designs, such as choke-rings or ground planes [19], is to identify or suppress reflected signals. However, multipath occurrence mainly depends on the roughness of the reflecting surface and the size of the active scattering region, which can be determined using the concept of Fresnel zones. That means, from a theoretical point of view, that only if the reflecting surface is smooth and large enough can multipath occur [20]. Since the majority of the aforementioned mitigation techniques deal with signals that are already affected by multipath, the antenna environment and, especially, the Fresnel zones are seldom taken into account.

Ray-tracing approaches use models of the antenna environment in order to analyze the signal paths between given satellite and antenna positions [21,22]. By determining the reflection points on the reflecting surfaces in the antenna surroundings, possible paths of reflected signals can be identified from a geometrical point of view. Hence, ray-tracing enables an assessment of the multipath level at certain positions. Furthermore, based on additional knowledge of the dielectric properties of the reflecting materials, observation corrections can also be derived. Although the antenna environment, in terms of 3D point clouds or virtual city models, is integrated into the multipath analysis, usually only single reflection points are considered in the ray-tracing algorithms and the active scattering regions are not taken into account.

One approach to minimize the influence of signal diffraction and NLOS reception is to replace the standard fixed-angle elevation mask with an azimuth-dependent elevation mask that represents the physical horizon from the antenna point of view. In [23], the azimuth-dependent elevation mask was determined using theodolite measurements and compared to a high fixed-angle elevation mask in mountainous regions. It was found that the azimuth-dependent elevation mask mitigates diffraction effects and leads to a higher positional accuracy, since the negative impact on the satellite geometry is lower compared to a high constant cut-off angle. In [24], the derivation of dynamic and azimuth-dependent elevation masks without terrestrial measurements directly from the measured SNR values was proposed. By comparing the SNR values to template functions generated under laboratory conditions, outliers can be erased and afterwards, the dynamic elevation mask can be determined by interpolating between the lowest elevations of the remaining SNR signals of the individual satellite tracks. The approach was tested in several urban scenarios, and it was shown that the quality of a network solution can be improved after application of the dynamic elevation masks to the carrier-phase observation. In [25], georeferenced 3D point clouds were used for the determination of obstruction adaptive elevation masks by extracting the upper borders of the obstruction sources in the antenna environment. After an adjustment of the elevation mask by considering the influence of the uncertainty of the initial antenna position, satellite signals that are subject to NLOS reception and signal diffraction were eliminated from the position estimation, leading to an improvement in the positional accuracy. However, Fresnel zones were not taken into account in any of the aforementioned approaches.

In recent years, attempts to use multipath as the signal of interest increased. Especially in the GNSS reflectometry (GNSS-R) community, ground reflected satellite signals are utilized in order to derive information, such as soil moisture content, snow depth or sea water levels [26,27]. In this context, Fresnel zones are used to identify and select the active scattering regions or sensing zones that contribute to the desired quantities. Furthermore, the Fresnel zones describe the spatial resolution of the GNSS-R experiments [28]. The basic idea is that the spectral content of signal-to-noise time series changes, if, for example, the soil moisture content changes in these regions [29]. The same holds for the sea level estimation. Here, the SNR time series of satellite signals, whose scattering regions are completely located on the water, are used to determine the height of the antenna above the water level [30]. In the context of GNSS-R, large Fresnel zones are preferred to increase the spatial sampling of the data retrieval. However, compared to the reflecting surface, such as open water or field area, the Fresnel zones are still small, and there is no doubt that the desired multipath effects will emerge.

In addition to the signal-to-noise ratio, the possibility of utilizing code-, carrier-phase or Doppler observations has been investigated in the context of GNSS-R applications such as sea surface topography, flood monitoring and tsunami detection [31,32]. Especially in the area of airborne altimetry, the huge number of available GNSS satellites could improve the spatiotemporal resolution compared to current radar altimeter missions [33]. Nevertheless, due to the high accuracy requirements, and to efficiently utilize reflected GNSS signals, these approaches often entail the need for a special antenna setup and receiver design [34]. Furthermore, the data processing is far more complex than for SNR-based GNSS-R applications. The Fresnel zone concept is applied in the same way as for ground-based GNSS-R applications, whereby the Fresnel zone dimensions can reach up to several kilometers and thus, are substantially larger than for ground-based experiments.

Contrarily, in positioning applications, multipath effects are the accuracy limiting factor and in these cases, it can be more important to avoid regions where, under a certain satellite constellation, significant multipath can be expected. Hence, it seems to be obvious that the theoretical prerequisites for multipath occurrence should be used for this purpose. In this context, we focus on two main aspects that are analyzed based on dedicated field tests:

1. Identification of diffracted satellite signals by integrating Fresnel zones into the concept of Obstruction Adaptive Elevation Masks (OAEM).
2. Analyzing the relationship between multipath occurrence and reflector size using Fresnel zones and simulations based on theoretical prerequisites for the special case of a spatially limited horizontal reflector.

The remainder of the paper is structured as follows: In Section 2, the theory on multipath and Fresnel zones is presented. Section 3 covers the investigations on diffraction identification with Fresnel zones and OAEMs, and in Section 4, the multipath analysis using Fresnel zones is described. A summary and an outlook on further investigations is given in Section 5.

## 2. Multipath Theory

In this section, the theoretical basics of GPS multipath propagation and Fresnel zones are presented, closely following the comprehensive and detailed descriptions documented in [18,35,36]. In Section 2.1, the process of multipath interference is explained and in Section 2.2, a method for the determination of the attenuation factor α is described. This is followed by an explanation of the Fresnel zone theory in Section 2.3 and the determination of reflection points in Section 2.4. Finally, theoretical prerequisites for multipath occurrence are defined in Section 2.5.

### 2.1. Multipath Propagation

**Figure 1:**
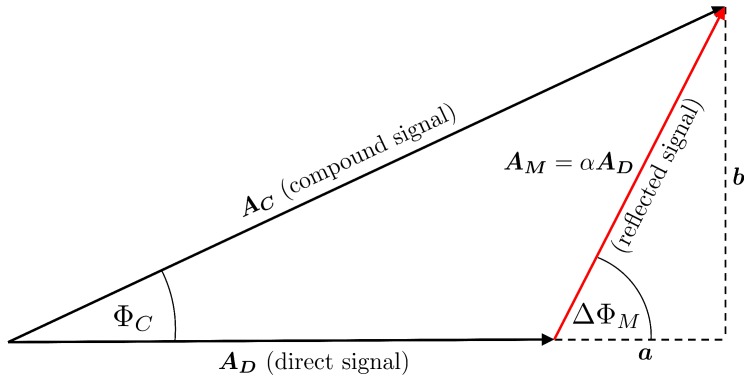
Resulting amplitude and phase error after superimposition of a direct signal and one reflected signal.

The superimposed multipath signal is composed by components of direct and reflected GNSS signals. This phenomena can be illustrated by means of a vector diagram, where the superimposed signal is the sum of two or more vectors representing the different signal components. Moreover, the length of the vectors denotes the amplitude of the respective signal components. In Figure 1, the respective vector diagrams for the superimposition of the direct signal and one reflected signal are shown.

The amplitude of the compound signal $A_{C}$ is the sum of the amplitudes of the direct signal $\left(A_{D}\right)$ and the reflected signal $\left(A_{M}\right)$, where $A_{M}=\alpha A_{D}$, with $\alpha =A_{M}/A_{D}$ representing the attenuation factor of the reflection process and the antenna gain pattern. $\Delta \Phi_{M}$ denotes the phase difference between the direct signal and the reflected signal due to the additional signal path and is called the multipath relative phase. The phase error resulting from the superimposition is denoted by $\Phi_{C}$.

The phase error between the direct and the compound signal $\Phi_{C}$ can be written as

(1) $$\tan\Phi_{C}=\frac{b}{A+a}$$

where the auxiliary values *a* and *b* can be derived from the multipath relative phase $\Delta \Phi_{M}$ and the amplitude $A_{M}$ by

(2) $$\begin{array}{c} a=A_{M}\cos\Delta \Phi_{M}=\alpha A_{D}\cos\Delta \Phi_{M}\\ b\;=A_{M}\sin\Delta \Phi_{M}=\alpha A_{D}\sin\Delta \Phi_{M}. \end{array}$$

After inserting Equation (2) into (1), the phase error of the compound signal $\Phi_{C}$ is given by

(3) $$\Phi_{C}=\arctan\left(\frac{\alpha \sin\Delta \Phi_{M}}{1+\alpha \cos\Delta \Phi_{M}}\right).$$

Using the amplitude $A_{D}$ and the expressions for *a* and *b*, the amplitude of the compound signal $A_{C}$ can be written as

(4) $$A_{C}^{2}={\left(A_{D}+\alpha A_{D}\cos\Delta \Phi_{M}\right)}^{2}+{\left(\alpha A_{D}\sin\Delta \Phi_{M}\right)}^{2}$$

and after rearranging Equation (4), $A_{C}$ can be determined by

(5) $$A_{C}=A_{D}\sqrt{1+2\alpha \cos\Delta \Phi_{M}+\alpha^{2}}.$$

Using Equation (3), the maximal and minimal phase errors can be estimated. Under the assumption that the reflected signal is not attenuated (α=1), the amplitude of the direct signal $A_{D}$ equals the amplitude of the reflected signal $A_{M}$. Thus, for $\Delta \Phi_{M}=180^{{}^{\circ}}$, the phase error $\Phi_{C}$ reaches its maximum of $90^{{}^{\circ}}$, which corresponds to one quarter of the respective signal wavelength (≈4.8 cm for GPS-L1). On the other hand, for $\Delta \Phi_{M}=0^{{}^{\circ}}$, the phase error becomes zero.

**Figure 2:**
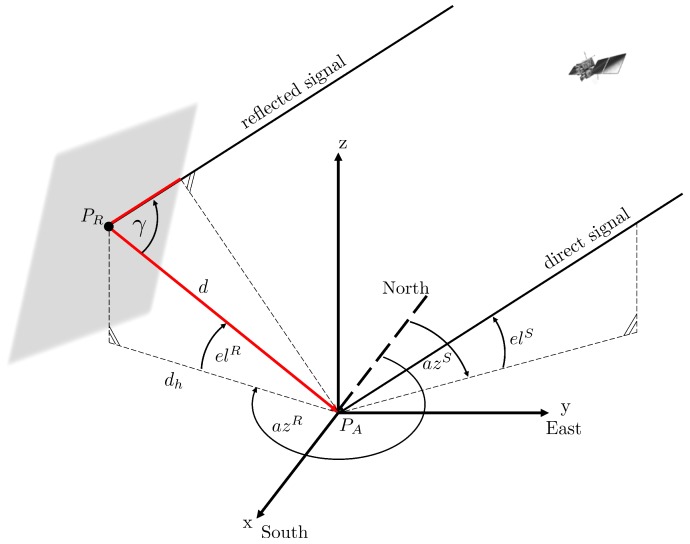
Geometrical satellite-reflector-antenna configuration for a single reflector with arbitrary orientation in space.

All of the parameters introduced above change over time and strongly depend on the geometrical configuration between the satellite, reflector and user antenna. In Figure 2, the geometrical configuration in a local horizontal coordinate frame is shown for a single reflector with arbitrary orientation in space.

Here, $az^{R}$ and $el^{R}$ denote the azimuth and elevation angles of the reflection point, and $az^{S}$ and $el^{S}$ denote the azimuth and elevation angles of the satellite, respectively. The distance between the antenna along the signal path of the reflected signal is denoted by *d*, and $d_{h}$ denotes the respective horizontal component.

Using the angle γ, the path delay δ (red part of the signal path) and the multipath relative phase $\Delta \Phi_{M}$ can be determined by

(6) $$\begin{array}{l} \;\delta \left(t\right)=d\left(1+\cos\gamma \left(t\right)\right)\\ \Delta \Phi_{M}\left(t\right)=\frac{2\pi }{\lambda }\delta \left(t\right)=\frac{2\pi d}{\lambda }\left(1+\cos\gamma \left(t\right)\right) \end{array}$$

where λ denotes the respective signal wavelength. Expressing γ as a function of the azimuth and elevation angles of the satellite and the reflection point leads to

(7) $$\Delta \Phi_{M}\left(t\right)=\frac{2\pi }{\lambda }\frac{d_{h}}{\cos el^{R}}\left[1-\cos el^{S}\left(t\right)\cos el^{R}\cos\left(az^{S}\left(t\right)-az^{R}\right)-\sin el^{S}\left(t\right)\sin el^{R}\right].$$

**Figure 3:**
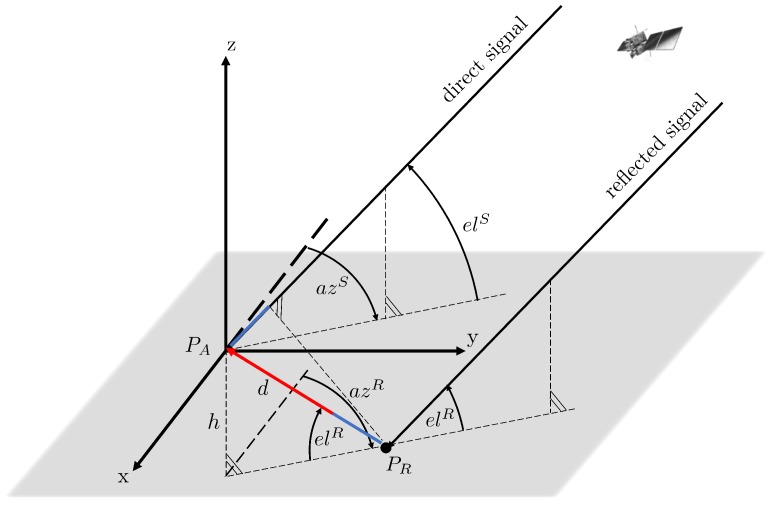
Geometrical satellite-reflector-antenna configuration for the special case of a single horizontal reflector.

In the special case of a horizontal reflector (see Figure 3), the satellite-reflection-antenna configuration simplifies. Here, the satellite and the points $P_{R}$ and $P_{A}$ form a vertical plane, leading to $az^{R}=az^{S}$. Furthermore, the elevation angle of the reflection point $el^{R}$ can be replaced by $-el^{S}$. Since the horizontal antenna-reflector distance $d_{h}$ changes due to the satellite motion, it can be replaced by a function of the satellite elevation $el^{S}$ and the vertical antenna-reflector distance *h*, as follows:

(8) $$d_{h}=\frac{h}{\tan el^{S}\left(t\right)}.$$

After integrating these simplifications into Equation (7), the geometrical path delay δ and the multipath relative phase can be expressed as

(9) $$\begin{array}{l} \;\delta \left(t\right)=2h\sin el^{S}\left(t\right)\\ \Delta \Phi_{M}\left(t\right)=\frac{2\pi }{\lambda }2h\sin el^{S}\left(t\right). \end{array}$$

### 2.2. Determination of the Attenuation Factor α

The attenuation factor α represents the ratio between the amplitudes of the reflected signal $A_{M}$ and the direct signal $A_{D}$ and can therefore also be denoted as a relative amplitude. Following Equations (3) and (5), α needs to be known for the determination of the phase error $\Phi_{C}$ and the amplitude of the compound signal $A_{C}$, respectively. Since both $A_{M}$ and $A_{D}$ cannot directly be measured by the receiver, instead, the provided signal-to-noise ratio (SNR) can be used to determine $A_{M}$ and $A_{D}$. Separating the direct and reflected signal strength components enables the determination of the relative amplitude.

The gain patterns of GNSS antennas are usually designed in such a way that signals received at low or negative elevation angles are attenuated. Hence, for ground reflected signals, it can be assumed that the amplitude $A_{M}$ is significantly lower than $A_{D}$. Thus, the dominant trend in SNR time series refers to the direct signal, and the multipath signals are modulated on top of this trend.

**Figure 4:**
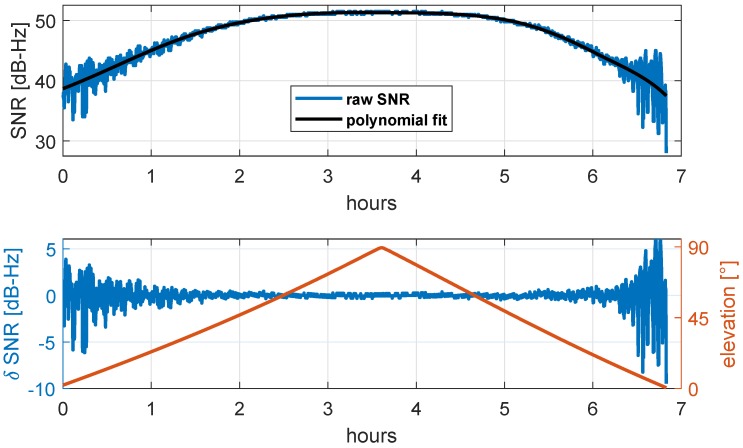
(**Top**) Raw signal-to-noise ratio (SNR) time series (blue) and polynomial fit (black) representing $A_{D}$. (**Bottom**) δ SNR (blue) representing $A_{M}$, after subtracting the polynomial fit from the raw SNR values, and related satellite elevation (red).

In Figure 4, an example for an SNR time series is shown.

The amplitude of the direct signal $A_{D}$ is modeled by fitting a polynom of lower order to the SNR time series, leading to the black curve shown in Figure 4. After subtracting $A_{D}$ from the SNR time series, the amplitude of the reflected signal $A_{M}$, especially at lower elevation angles, becomes more apparent. Finally, the epochwise attenuation factor $\alpha \left(t\right)$ can be determined by

(10) $$\alpha \left(t\right)=\frac{A_{M}}{A_{D}}=\frac{\delta SNR}{A_{D}}.$$

Alternatively, α can also be determined from the antenna gain pattern and the reflection coefficients of the reflecting material. Since the gain pattern is not available for the antennas used in the investigations presented here, this approach is not explained in detail. For further information, refer to [36].

### 2.3. Fresnel Zones

**Figure 5:**
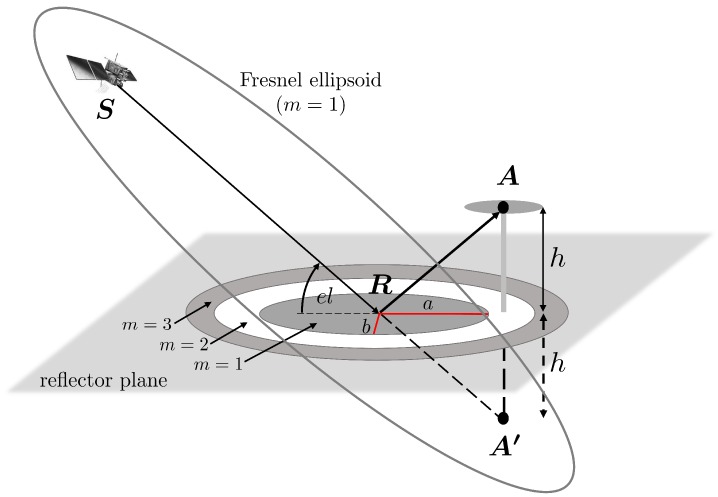
Intersection of Fresnel ellipsoid of the first order with the horizontal reflector plane.

During the derivation of the formulas for the determination of the phase error $\Phi_{C}$ and as shown in Figure 2 and Figure 3, only a single reflection point is considered. However, GPS satellites are transmitting the signals with an aperture angle of $\pm 13.9^{{}^{\circ}}$ and thus, there are existing areas on the reflector surface that are contributing to the reflected signal. These active scattering regions are called Fresnel zones. The concept of Fresnel zones is illustrated in Figure 5.

**Figure 6:**
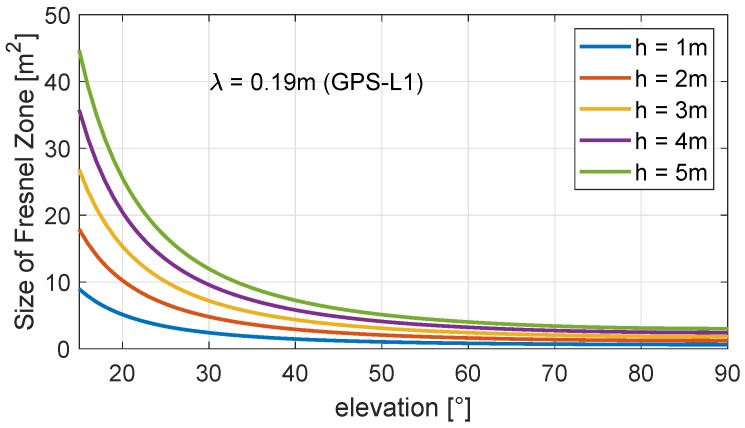
Size of Fresnel zone for different satellite elevation angles and antenna heights determined for the GPS-L1 carrier wavelength.

Between the satellite S and the mirrored antenna point $A^{\prime }$, a number (orders *m*) of ellipsoids of revolution can be formed, with S and $A^{\prime }$ representing the respective focal points [37]. The Fresnel ellipsoid of first order defines the region where most of the energy is transmitted. The intersection of the Fresnel ellipsoids and the reflector surface is called a Fresnel zone. In the case of a horizontal reflector, the size and shape depends on the satellite elevation angle el, the carrier wavelength λ and the vertical distance *h* between the antenna and the reflector. The semi-major and semi-minor axes of the first Fresnel zone are given by

(11) $$\begin{array}{c} b=\sqrt{\frac{\lambda h}{\sin el}+{\left(\frac{\lambda }{2\sin el}\right)}^{2}}\\ a=\frac{b}{\sin el} \end{array}$$

and the orientation of the major axis is defined by the direction of the satellite-antenna vector [38]. In Figure 6, the size of the first Fresnel zone is shown for satellite elevation angles between $15^{{}^{\circ}}$ and $90^{{}^{\circ}}$ and antenna heights of 1 to 5 m.

For a horizontal reflector, the size of the Fresnel zone increases as the elevation angle decreases, and it reaches its minimum at an elevation angle of $90^{{}^{\circ}}$. For an antenna height of h=1 m, this leads to F≈0.6 m^2^ and for h=5 m, this leads to F≈3 m^2^. Furthermore, the elevation angle determines the eccentricity of the ellipse (see Equation (11)). Thus, the Fresnel zone has a circular shape if the elevation angle is $90^{{}^{\circ}}$, and it is stretched for decreasing elevation angles.

**Figure 7:**
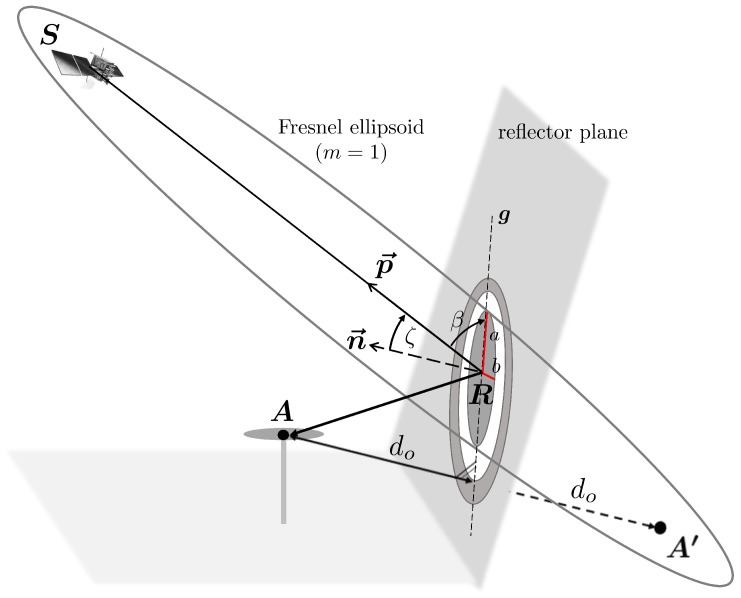
Intersection of Fresnel ellipsoid of the first order with an arbitrarily oriented reflector plane.

For vertical reflectors, or, in general, for reflectors with arbitrary orientation in space, as shown in Figure 7, the parameters of the Fresnel zones, *a* and *b*, cannot directly be determined following Equation (11).

Since the satellite elevation angle el no longer equals the angle between the signal and the reflector plane, it has to be substituted. For this purpose, at first, the incidence angle ζ of the satellite signal with respect to the reflector plane is determined by

(12) $$\zeta =\arccos\left(\frac{\vec{n}\cdot \vec{p}}{∥\vec{n}∥\cdot ∥\vec{p}∥}\right)$$

where $\vec{n}$ denotes the normal vector of the reflector plane and $\vec{p}$ is the vector between the satellite S and the reflection point R. Afterwards, the angle β can be expressed as

(13) $$\beta =90^{{}^{\circ}}-\zeta .$$

Moreover, the antenna height *h* is substituted by the orthogonal distance between the antenna and the reflector plane $d_{o}$, leading to

(14) $$\begin{array}{c} b=\sqrt{\frac{\lambda d_{o}}{\sin\beta }+{\left(\frac{\lambda }{2\sin\beta }\right)}^{2}}\\ a=\frac{b}{\sin\beta }. \end{array}$$

The orientation of the major axis *a* of the Fresnel zones is given by the orientation of the line, resulting from the intersection of the reflector plane and a plane defined by S, A and $A^{\prime }$. Using Equation (14), the Fresnel zones can be determined for every planar reflector, without any limitations regarding the satellite-reflector-antenna configuration.

Generally, for arbitrarily oriented reflectors, valid statements regarding the size of the Fresnel zones are not possible, since, in this case, the size depends on both the orientation of the reflector and the satellite azimuth and elevation angles.

**Figure 8:**
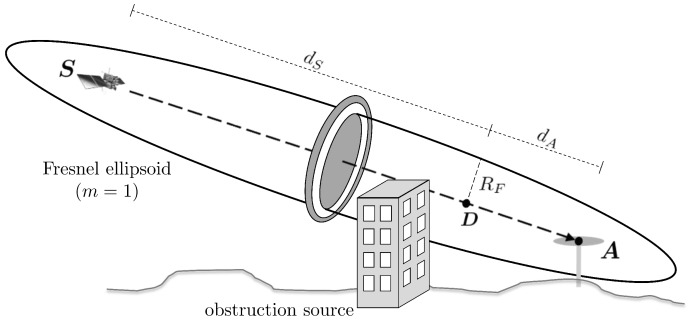
Fresnel ellipsoid of the first order for line-of-sight transmission between the satellite S and the antenna A including a building obstructing the signal path and parts of the first Fresnel zone.

In addition to determining Fresnel zones on the reflecting surface, the concept can also be considered for the line-of-sight (LOS) transmission in order to characterize the type of signal propagation [20]. In Figure 8, the Fresnel ellipsoid of the first order is shown for the LOS transmission between the satellite S and the antenna A.

The radius $R_{F}$ of the Fresnel zone at any point D along the signal path can be determined by

(15) $$R_{F}=\sqrt{\frac{\lambda d_{S}d_{A}}{d}}$$

where λ is the carrier wavelength, $d_{S}$ denotes the distance between *D* and *S*, $d_{A}$ denotes the distance between *D* and *A*, and $d=d_{S}+d_{A}$ is the total length of the signal path [37]. An obstruction source in the signal path with larger dimensions than the Fresnel zone will lead to a complete blocking of the signal. If the obstruction source in the signal path is smaller than the Fresnel zone, the signal can be diffracted at the edges of the object. Without any obstruction source, the signal transmission will be undisturbed.

**Figure 9:**
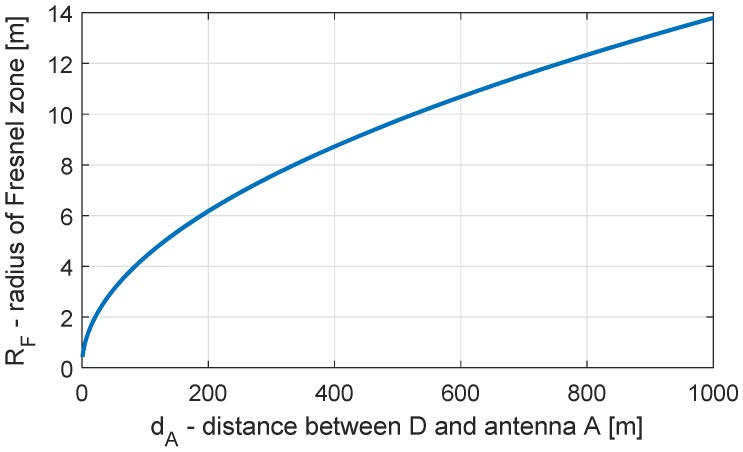
Radius of Fresnel zone for line-of-sight (LOS) transmission.

In order to get an impression of the dimensions of the Fresnel zones along the signal path, $R_{F}$ is shown in Figure 9 for $d_{A}=1\ldots 1000$ m and λ=0.19 m (GPS-L1). The total length of the signal path is set to d= 20,000 km.

It becomes obvious that, especially for satellites with lower elevation angles, small objects in the environment can lead to diffraction effects. Thus, Figure 9 emphasizes that the choice of a proper elevation mask is an important step during data processing.

### 2.4. Determination of Reflection Points via Ray-Tracing

The determination of the Fresnel zones requires knowledge on the coordinates of the reflection point R.

**Figure 10:**
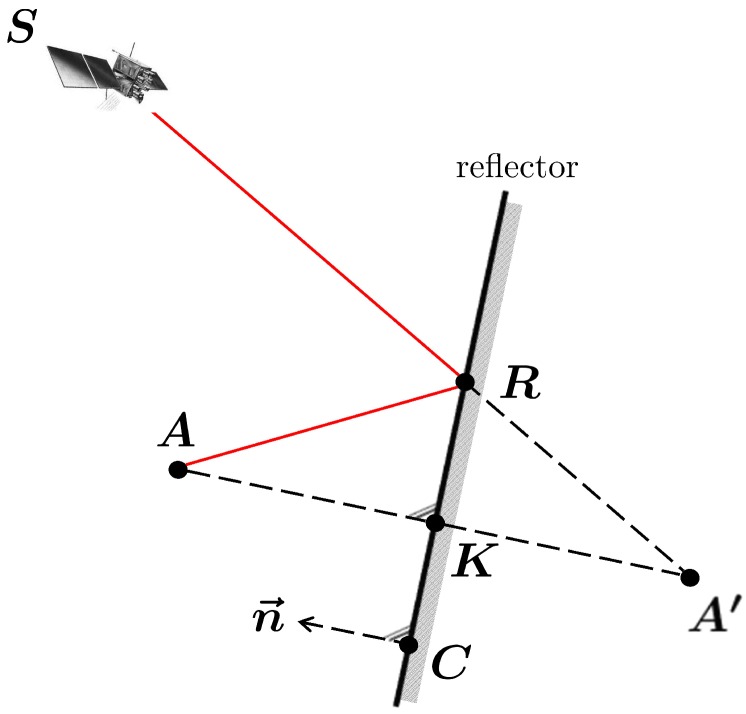
Ray-tracing for determination of reflection point *R*.

Therefore, point K is determined by projecting the antenna position A onto the reflector plane along the direction of the respective normal vector $\vec{n}$ by

(16) $$K=A+\frac{\left(C-A\right)\cdot \vec{n}}{\vec{n}\cdot \vec{n}}\vec{n}$$

where C denotes an arbitrary point on the reflector plane (cf. Figure 10). Afterwards the mirrored antenna position $A^{\prime }$ is determined by

(17) $$A^{\prime }=A+2\left(K-A\right).$$

Finally, the reflection point R can be computed by intersecting the reflection plane and the line and joining the satellite position S and the mirrored antenna position $A^{\prime }$ by

(18) $$R=A^{\prime }+\frac{\left(C-A^{\prime }\right)\cdot \vec{n}}{\left(S-A^{\prime }\right)\cdot \vec{n}}\left(S-A^{\prime }\right).$$

### 2.5. Theoretical Prerequisites for Multipath Occurrence

**Figure 11:**
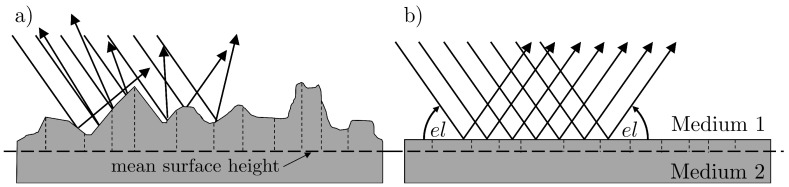
(**a**) Diffuse reflection at a rough surface. (**b**) Specular reflection at a smooth surface. In (**a**) and (**b**), the horizontal dashed line represents the mean surface height, and the vertical dashed lines are the differences from the mean surface height.

In order to formulate theoretical prerequisites for multipath occurrence, it is necessary to distinguish between *diffuse* and *specular* reflections. In Figure 11, both types of reflection are illustrated schematically.

Basically, the type of reflection depends on the signal wavelength λ, the satellite elevation angle el and the roughness ΔH of the reflecting surface, where roughness is usually described as the standard deviation of the difference to a mean surface height. With these values, the *Rayleigh criterion*,

(19) $$\Delta H\leq \frac{\lambda }{8\sin el},$$

can be formulated and used to decide whether diffuse or specular reflection will occur. If the Rayleigh criterion is not fulfilled, the reflecting surface will lead to diffuse reflection, i.e., the signal will be scattered in different directions. Hence, this type of reflection has a rather random nature and can be denoted as less critical. Contrarily, if the Rayleigh criterion is fulfilled, i.e., the surface is rather smooth compared to the signal wavelength, mostly specular reflection will occur, leading to the phenomena described in Section 2.1. In Table 1, the ΔH-values for different satellite elevation angles and the GPS-L1 carrier wavelength (λ=0.19 m) are listed.

Table 1 shows that the occurrence of specular reflection is more likely for lower elevation angles, since the surface roughness ΔH can be bigger compared to higher elevation angles. Especially for the first Fresnel zone, the Rayleigh criterion should be taken into account. As described in Section 2.3, in this region, most of the signal energy will be reflected and thus, the surface roughness in this area is significant for distinguishing between diffuse and specular reflection. Therefore, as a first prerequisite for multipath occurrence, the reflecting surface should fulfill the Rayleigh criterion defined by Equation (19), or in other words, the reflecting surface has to be smooth enough compared to the signal wavelength.

The second prerequisite is related to the size and location of the Fresnel zones with respect to the reflecting surface. Since the first Fresnel zone defines the region where most of the signal energy is reflected, at the same time, it defines the minimum size of the reflector. That means that for multipath to occur, the reflector needs to be larger than the first Fresnel zone, so that a sufficient amount of energy is reflected, and the Fresnel zone needs to be completely located on the reflecting surface

## 3. Identification of Diffracted Satellite Signals Using Fresnel Zones and OAEMs

Obstruction Adaptive Elevation Masks can be derived from georeferenced models of the antenna environment, such as point clouds from terrestrial laser scanners (TLS) [25]. After determining the azimuth and elevation of all points and allocating them to an azimuthal grid, the OAEM is obtained by selecting the highest elevation angle in each grid cell. From the viewpoint of the antenna, the OAEM separates obstructed areas from open sky and can be used to identify satellite signals that are subject to NLOS reception or signal diffraction, leading to a substantial improvement in the positioning accuracy. However, in this algorithm, only the direct line-of-sight (LOS) directions of the satellite signals are analyzed and the Fresnel zones along the signal path (cf. Figure 8) are not taken into account.

For this purpose, in Section 3.1, the integration of Fresnel zones into the OAEM algorithm is presented. Based on the data of a field test described in Section 3.2, the algorithm is assessed in Section 3.3.

### 3.1. Identification Algorithm

The identification algorithm uses the OAEM as an representation of possible obstructions sources in the surroundings of the antenna. For each cell $c_{i}$ of the OAEM, the respective elevation angle $el_{i}$ and the topocentric coordinates of the related TLS scan point, $p_{i}=\left[\begin{array}{ccc} e_{i} & n_{i} & u_{i} \end{array}\right]$, are stored. If cells are empty because there are no obstruction sources in this azimuthal direction, $el_{i}$ is set to a predefined standard value, e.g., $5^{{}^{\circ}}$, and the coordinates of $p_{i}$ are determined by linearly interpolating between the coordinates of the next adjacent and filled cells.

**Figure 12:**
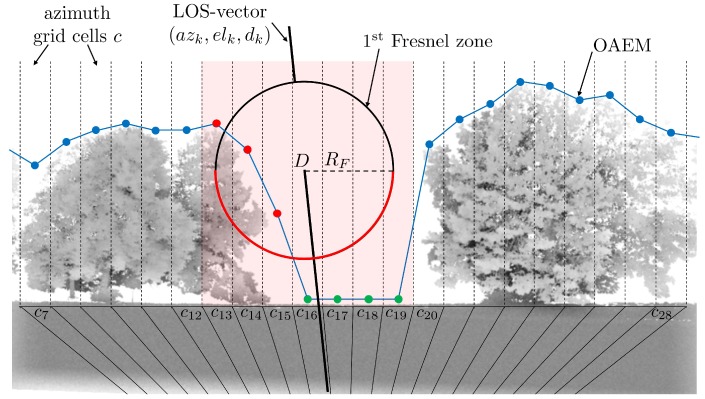
Diffraction identification using the Fresnel zone. The Obstruction Adaptive Elevation Mask (OAEM) is represented by the elevation angles (blue dotted line) and the respective azimuth grid cells *c*. The half red and half black circle represent the first Fresnel zone with radius $R_{F}$ determined for point *D* on the direct signal path along the LOS-vector, which is represented by the azimuth, elevation and distance to satellite *k*. The red shaded cells indicate an overlap with the Fresnel zone, and the red and green dots denote whether the elevation angles of points on the red semicircle are lower than the OAEM or not.

In Figure 12, the basic features of the algorithm are illustrated. It should be noted that since the proposed algorithm is identical for every satellite *k* and every observation epoch *t*, in the following description of the algorithm, the indices *k* and *t* are omitted for reasons of clarity.

**Figure 13:**
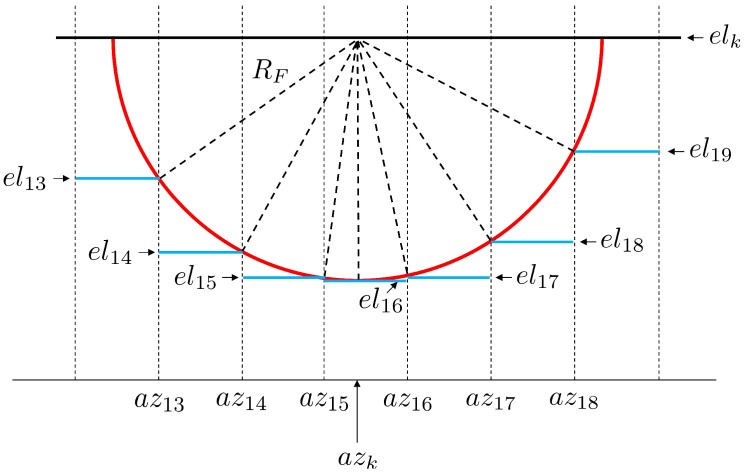
Enlarged display of the Fresnel zone and azimuth cell grid from Figure 12. The blue lines denote the elevation angles for the intersection points of the Fresnel zone boundary (red line) and cell borders (vertical dotted lines). The satellite azimuth angle is denoted by $az_{k}$, and $R_{F}$ denotes the Fresnel zone radius.

For the identification or exclusion of diffracted signals, in a first step, the grid cell $c_{i}$ that contains the satellite azimuth angle az ($c_{16}$ is identified, as shown in Figure 12). Afterwards, the radius $R_{F}$ of the first Fresnel zone is determined at position $p_{i}$ following Equation (15). After $R_{F}$ has been converted to the angular value $\alpha_{R}$, the azimuth angles of all points of the Fresnel zone are inside the interval $az_{F}=\left\{az-\alpha_{R},az+\alpha_{R}\right\}$. At the same time, all grid cells $c_{j}$ that intersect with the Fresnel zone are derived from $az_{F}$ ($c_{13}\ldots c_{19}$). In order to identify the obstructed areas of the Fresnel zone, it is sufficient to test if any part of the lower semicircle of the Fresnel zone boundary is lower than the elevation angles in the related grid cell. In Figure 13, an enlarged display of the relevant part of Figure 12 is shown.

It becomes obvious that only the point with the lowest elevation angle on the Fresnel zone boundary needs to be identified in each cell. As shown in Figure 13, these points are the intersection points of the cell borders and the Fresnel zone boundary. Thus, by applying the law of Pythagoras, for each cell $c_{j}$, one elevation angle $el_{j}$ is determined by

(20) $$el_{j}=\left\{\begin{array}{cc} el_{k}-\sqrt{R_{F}^{2}-{\left[\left(az_{j-1}-az_{k}\right)\cdot d\right]}^{2}}, & j>i,\\ el_{k}-\alpha_{R}, & j=i,\\ el_{k}-\sqrt{R_{F}^{2}-{\left[\left(az_{j}-az_{k}\right)\cdot d\right]}^{2}}, & j<i, \end{array}\right.$$

where *d* denotes the distance between the antenna and $p_{i}$, and the elevation and azimuth angles are given in radians.

In the last step, the elevation angles $el_{j}$ are compared to the respective values in the OAEM. If in any of the grid cells $el_{j}$ is smaller than the OAEM value, parts of the Fresnel zones are obstructed, and although the direct signal path is not blocked, diffraction effects can be expected (cf. Section 2.3). In Figure 12, this is the case for cells $c_{13}$ to $c_{15}$.

### 3.2. Measurement Concept

**Figure 14:**
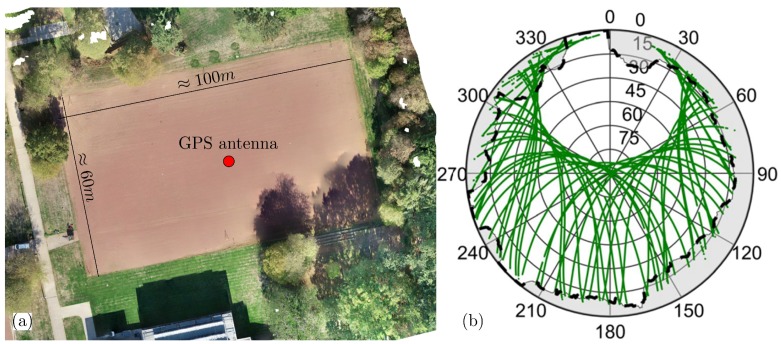
(**a**) Orthophoto of the test area from a UAV flight. The red point marks the location of the GPS antenna on the cinder pitch. (**b**) Obstruction adaptive elevation mask (black dashed line) of test area determined from a georeferenced terrestrial laser scanner (TLS) point cloud. The green lines show the GPS satellite tracks during the 24 h observation period.

The field test was performed on a cinder pitch, as shown in the left panel of Figure 14. The pitch has dimensions of 60 times 100 m and is surrounded by trees in several directions. Additionally, one building can be found in the Southern direction.

For about 24 h, one Leica AS10 GNSS antenna in combination with a Leica GS25 receiver was mounted on a tripod. The antenna was located approximately in the middle of the pitch (c.f. red dot in Figure 14), and dual frequency GPS observations were logged at a sampling rate of 0.2 Hz. Furthermore, the Geodetic Post-Processing-Service (GPPS) of the German Satellite-Positioning-Service (SAPOS) (https://www.sapos.de) were used to generate GNSS observations of a virtual reference station (VRS) for the complete duration of observations. To establish a short baseline between the GNSS antenna and the VRS, the VRS was located at the navigation solution of the antenna.

Prior to the GPS measurements, a UAV flight was performed, and the area was captured with a Leica ScanStation P20 panoramic-type laser scanner. Both the processed UAV images and the TLS pint cloud were georeferenced using ground control points, which were determined via GPS-RTK.

Finally, the georeferenced point cloud was used to generate the obstruction adaptive elevation mask for the test area following the procedure described in [25]. In the right panel of Figure 14, a skyplot including the OAEM and the GPS satellite tracks is shown.

### 3.3. Algorithm Assessment

The algorithm assessment was based on the analysis of the coordinates from a kinematic baseline solution. For this purpose, the original data set was modified in two different ways:

1. All satellite signals with an elevation angle lower than the respective OAEM values were excluded (hereafter denoted as *classical OAEM*).
2. In addition to the signals detected by the OAEM, all signals that were identified by the proposed algorithm as being potentially influenced by diffraction effects were also excluded (hereafter denoted as *OAEM with Fresnel zones*).

For all data sets, a kinematic baseline solution was carried out. In each case, the VRS served as the master station. The estimation of the baseline parameters is performed in the three steps: (1) float solution, (2) integer ambiguity fixing and (3) fixed solution. In the float solution step, the ambiguities were estimated as real numbers. Afterwards, the float ambiguities and their covariance matrix were used to fix the the ambiguities to integer values using the LAMBDA method [39]. The result of the fixing process was validated using the ratio test [40]. Herein, the squared norm of the residuals of the best set of ambiguities ($R_{1}$) and the second best set of ambiguities ($R_{2}$) were compared. If the quotient $R_{2}/R_{1}$ exceeded a predefined threshold of 3, the ambiguity fixing was accepted. In the final step, the fixed solution, the baseline parameters were estimated after the fixed ambiguities had been added to the observations and were eliminated from the parameter vector.

**Figure 15:**
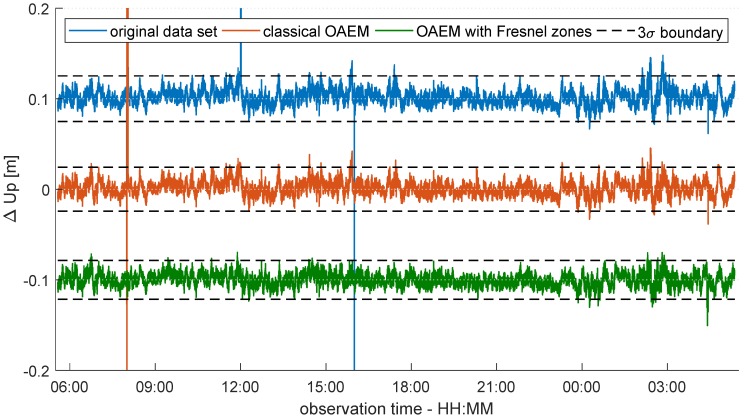
Differences in the Up-component for the original data set (blue), the data set modified by the classical OAEM approach (red) and the data set modified by the newly proposed algorithm (green). For better visualization, the blue and green curves are shown with offsets of 10 and −10 cm respectively. Differences exceeding the axis limits are truncated. The black dashed line represents the 3σ boundary of each time series.

Afterwards, for better visualization purposes, the resulting baseline vectors were transformed to a local topocentric coordinate system (East, North, Up), and the coordinates were reduced by the results of a static batch solution from a commercial software package (Leica Infinity (https://leica-geosystems.com/en-US/products/gnss-systems/software/leica-infinity)). In Figure 15, the coordinate differences of the up-component and the respective 3σ boundaries are shown exemplarily for each data set. Additionally, Table 2 lists the minimum and maximum coordinate differences and the root-mean-squared error (RMS) of the time series. Furthermore, the percentage of outliers with respect to the 3σ boundary, as well as the percentage of observation epochs in which the carrier phase ambiguities could be fixed to integer values are listed.

Both the coordinate differences shown in Figure 15 and the related values listed in Table 2 indicate that using the Fresnel zone concept for detecting and excluding diffracted signals can lead to an improvement of the coordinate accuracy. After the classical OAEM approach has been applied to the data set, the percentage of epochs with successfully fixed ambiguities increases. Although in two cases (cf. 12:00 and 16:00 in Figure 15) the highest differences in the original data set can be reduced, one significant peak remains (cf. 08:00 in Figure 15). Only after integrating the Fresnel zones can the number of fixed solutions be further increased, and consequently the coordinate differences vary between the maximum and minimum values of 3 cm and −5 cm, respectively. In contrast, the RMS values of the time series are similar for every data set. This is reasonable, since the satellite signals where the Fresnel zone partially intersects the obstruction source (cf. Figure 12) will not necessarily have a significant impact on the coordinate estimation. Nevertheless, compared to the results from the classical OAEM approach, the number of differences that exceed the 3σ boundary is reduced, which corresponds to the lower peaks in some observation epochs that are only present in the red coordinate time series in Figure 15. Hence, especially in applications, such as deformation monitoring or kinematic positioning, where accurate coordinates are needed in every observation epoch, the proposed algorithm could exploit its full potential.

## 4. Multipath Analysis from a Spatially-Limited Reflector Using Fresnel Zones

Theoretically, multipath effects can only occur if the reflecting surface meets the following two prerequisites (cf. Section 2.5):

1. The reflecting surface needs to be smooth enough. That means, the Rayleigh criterion defined by Equation (19) is fulfilled.
2. The reflecting surface needs to be large enough. That means, the first Fresnel zone should completely be located on the reflecting surface.

The aim of this section is to empirically analyze and assess these theoretical assumptions using the data from an appropriate field test in a controlled environment. The measurement concept and the data preparation are described in Section 4.1. Afterwards, in Section 4.2, the multipath analysis is presented, which is performed by comparing real SNR observations to simulations that are derived from the multipath propagation theory (cf. Section 2) and by relating the results to the location and size of the respective Fresnel zones.

### 4.1. Measurement Concept and Data Preparation

Empirical investigations on the relationship between the location and size of the Fresnel zones and the reflecting surface are not easy to realize. Generally, separating the multipath effects from several reflectors is a non-trivial problem, and thus, it seems to be straightforward to reduce the problem on one single reflecting surface—the ground. However, since the ground is usually not spatially limited, it is difficult to answer the question of whether multipath effects will occur even though the Fresnel zones are not fully located on the reflector.

**Figure 16:**
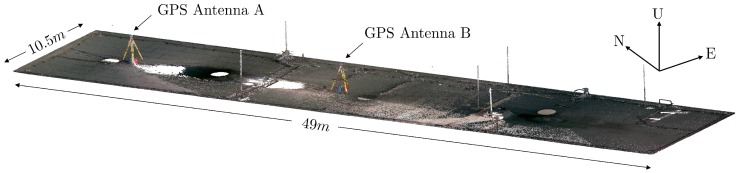
Colored point cloud of the building roof and the positions of GPS antennas A and B.

Therefore, the field test was carried out on the roof of a building, which, due to its exposed position, was the only dominant reflector in the antenna environment and at the same time, was spatially limited by the roof edges. Due to security and privacy reasons, a UAV flight was not possible, and instead of an aerial image, a colored point cloud of the roof is shown in Figure 16. The point cloud was created using a Leica BLK360 imaging laser scanner and was georeferenced using control points determined via GPS-RTK.

The roof has dimensions of 49 times 10.5 m and is covered by roofing felt. Although the roof is the highest point in the surroundings, it is not an completely ideal reflector. The six thin lightning rods shown in Figure 16 could not be removed during the measurements, since the metal wires were attached to a few small concrete brackets along the roof edges. Nevertheless, it can be assumed that their influence on the measurements should be small compared to the multipath effects. Furthermore, their locations are known and it should be possible to trace back anomalies in the observations that can not be explained by ground reflections.

For about 24 h, two Leica AS10 GNSS antennas (A and B) in combination with Leica GS25 receivers were mounted on tripods with the same antenna height of 1.84 m and were placed at the positions shown in Figure 16. At both stations, dual frequency GPS observations were logged at a sampling rate of 0.2 Hz. After the measurements, the raw observations were synchronized between the two antennas using the GPS time stamps in the respective RINEX files, and the azimuth and elevation angles for all observed satellites were determined from the broadcast ephemeris.

It should be noted that prior to the field test, both antennas were individually calibrated for their phase center offset and variations in the anechoic chamber of the University of Bonn [41]. Accounting for the height difference between the antenna reference point and the antenna phase center is an essential step during the simulation described in the next section.

### 4.2. Multipath Analysis

In preparation for the multipath analysis, the smoothness of the reflecting surface was determined. Therefore, a plane was fitted to the TLS point cloud of the roof and the standard deviation $\sigma_{\Delta H}$ of the residuals was computed as $\sigma_{\Delta H}=0.055$ m. By inserting $\sigma_{\Delta H}$ into Equation (8), the corresponding elevation angle was determined, leading to $26^{{}^{\circ}}$ for the GPS-L1 carrier phase wavelength. That means that for satellite signals with elevation angles lower than $26^{{}^{\circ}}$, specular reflection could be expected. However, the Rayleigh criterion should not be interpreted as a sharp border between specular and diffuse reflection [18], but in the subsequent multipath analysis, it should be taken into account that the propability for diffuse scattering increases for signals with higher elevation angles.

**Figure 17:**
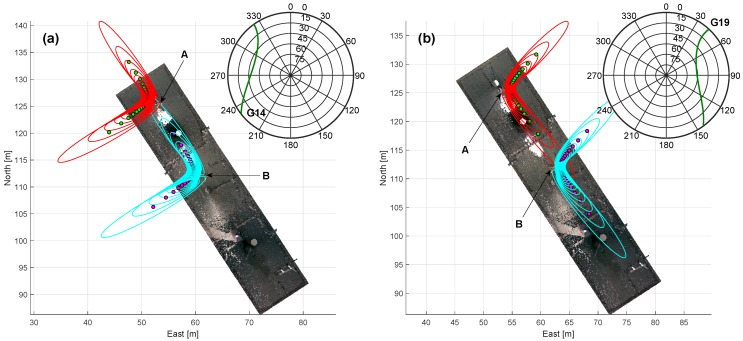
Fresnel zones and reflection points related to antenna A (red ellipses and green dots) and B (cyan ellipses and magenta dots) for satellites G14 (**a**) and G19 (**b**) in a topocentric coordinate system. The Fresnel zones are shown for every hundreth observation epoch. Top right in left and right figures: skyplot with satellite tracks of G14 and G19, where the PRN number is located at the end of each track.

In the first step of the multipath analysis, for every satellite and every observation epoch, the reflection points were determined according to Equations (16)–(18). Herein, the required normal vector of the reflector plane **n** was estimated during a plane fit in the Gauß–Helmert model [42]. Afterwards, the semi-axes and the orientation of the Fresnel zones were computed from Equation (14). In Figure 17, the Fresnel zones and the respective reflection points of every hundredth epoch are shown for both antennas in a topocentric coordinate system for satellites G14 and G19, exemplarily. Furthermore, the satellite tracks of G14 and G19 are visualized in a skyplot.

On the one hand, Figure 17 visualizes the theoretical considerations from Section 2.3. The Fresnel zones (red and cyan lines) form ellipses in the reflector plane with the reflection points (green and magenta dots) located in the middle of the ellipses. Depending on the elevation of the satellite signal, the shape of the ellipse becomes elongated, and the reflection points move away from the antenna. Furthermore, it becomes obvious that for horizontal reflection planes, the orientation of the Fresnel zones is defined by the azimuth angle of the respective satellite.

On the other hand, the Fresnel zones of satellites G14 and G19 illustrate the basic idea of the experiment. Satellite G14 rises at an azimuth angle of approximately $320^{{}^{\circ}}$, passes both antennas in the Western direction and sets at an azimuth of around $235^{{}^{\circ}}$. Due to the spatially limited and rectangular shape of the roof, Fresnel zones are only completely located on the roof for certain directions and elevations. For antenna B, this is especially the case during the rising phase of G14. Since antenna A is placed nearer to the edge of the roof, the Fresnel zones determined for the same satellite positions are only partially located on the roof or they are completely disjoint. Satellite G19 rises at an azimuth of $145^{{}^{\circ}}$ and passes the antennas in the Eastern direction before it sets at an azimuth of $45^{{}^{\circ}}$. In this case, the location of the Fresnel zones with respect to the reflecting surface are nearly identical, and the multipath analysis should lead to similar results.

Thus, for each antenna, it becomes possible to analyze the relationship between the size and location of the Fresnel zones and multipath occurrence. For this purpose, SNR observations are used, since they reflect the characteristics of multipathed signals, especially after the trend from the direct signal has been removed (cf. Section 2.2).

**Figure 18:**
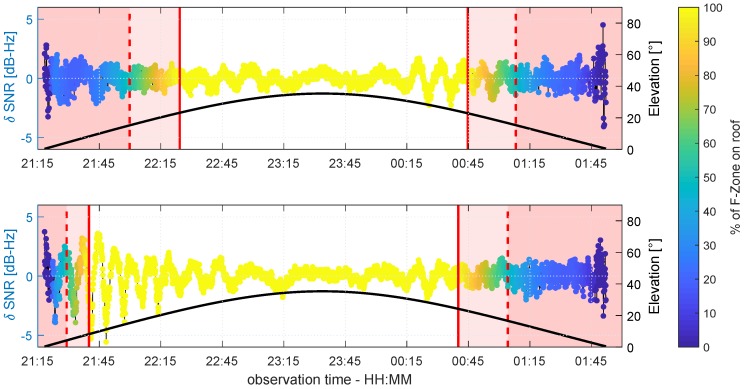
Color coded δ-SNR time series of G14 observed at antenna A (**top** panel) and antenna B (**bottom** panel). The black line shows the elevation angle of G14 during the observation period.

In Figure 18, the δ-SNR time series of G14 is shown for antennas A and B. The values are color coded according to the percentage $P_{FZ}$ of overlap between the Fresnel zone and the roof. Between the solid red lines, both, the Fresnel zones and the reflection points are completely located on the roof ($P_{FZ}=100\%$). The pink shaded areas bounded by the solid and dashed red lines represent the transition phases, where the reflection point and only parts of the Fresnel zones are located on the roof ($50\%\leq P_{FZ}\geq 100\%$). In the red shaded areas (left and right of the dashed red lines), the reflection point is not located on the roof and $P_{FZ}\leq 50\%$.

It can be noticed that during the rising phase of G14, the time from which the Fresnel zones are completely located on the roof appears earlier at antenna B (≈21:40) than at antenna A (≈22:25). Furthermore, the typical multipath oscillations occur in these 100% sections, most visibly between 21:45 and 22:45 at antenna B. In this period, the elevation angle is below $30^{{}^{\circ}}$, and thus, corresponds with the considerations on the Rayleigh criterion at the beginning of this section. Contrarily, in the red shaded areas at the beginning and end of the time series, the systematic and periodic behavior disappears and the curve shape is of a more random nature. This is reasonable, since the reflection points are not located on the roof, and the overlap of the Fresnel zones and the roof is only up to 50%. Hence, if only these two regions are taken into account, the relationship between the Fresnel zone concept and multipath occurrence can be confirmed. Nevertheless, the most interesting period of time is the transition phase ($50\%\leq P_{FZ}\geq 100\%$), represented by the pink shaded region, where the reflection points are still on the roof, but the Fresnel zones are only partially overlapping the reflector. During the rising phase of G14, the δ-SNR time series of antenna A shows more random behavior in this region. In comparison, in the setting phase of G14, the multipath oscillations are still visible but with a slightly lower amplitude. The same holds for antenna B but in reverse order. Here, the oscillations are clearly visible during the rising phase of G14.

**Figure 19:**
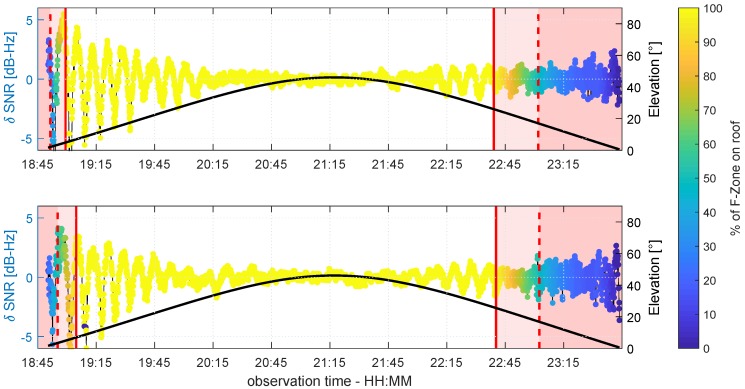
Color coded δ-SNR time series of G19 observed at antenna A (**top** panel) and antenna B (**bottom** panel). The black line shows the elevation angle of G19 during the observation period.

In Figure 19, the same data is shown for satellite G19.

Due to the orientation of G19 with respect to the roof, the three phases of $P_{FZ}$ are similar at both antennas, and the δ-SNR curves show the same behavior. During the rising phase, G19 moves according to the orientation of the roof and only at the lowest elevation angles of around $5^{{}^{\circ}}$ are the Fresnel zones not completely located on the roof. As a consequence, the transition phase and the phase where $P_{FZ}\leq 50\%$ are very short, and the shape of the δ-SNR time series cannot clearly be analyzed. During the complete overlapping of the Fresnel zones and the roof, the characteristic multipath oscillations appear at both antennas, whereby the amplitudes are of the same order of magnitude. From the beginning of the transition phase until the end of the time series, the systematic effects disappear, and from 23:00 onwards, the shape of both curves is of a random nature.

**Figure 20:**
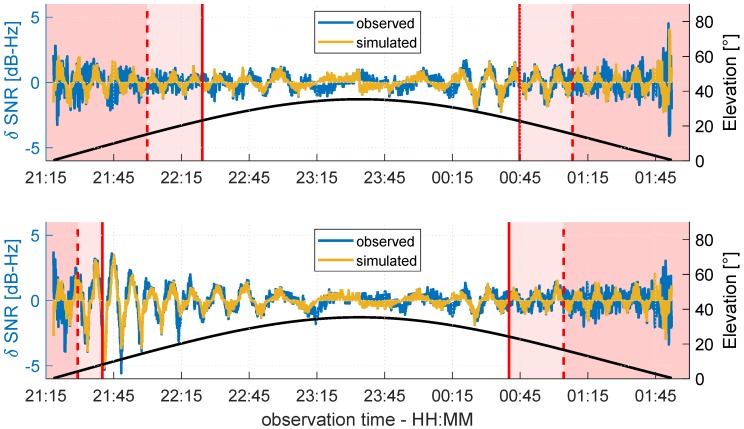
Observed (blue) and simulated (ocher) δ-SNR time series of G14 for antenna A (**top** panel) and antenna B (**bottom** panel). The black line shows the elevation angle of G14 during the observation period.

**Figure 21:**
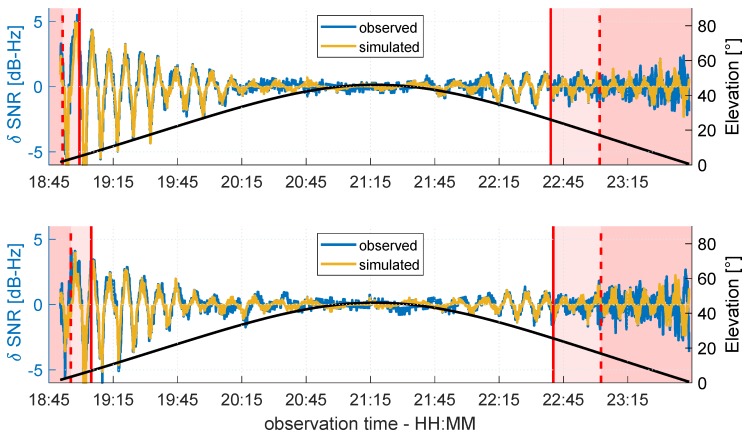
Observed (blue) and simulated (ocher) δ-SNR time series of G19 for antenna A (**top** panel) and antenna B (**bottom** panel). The black line shows the elevation angle of G19 during the observation period.

In order to better assess the accordance between the real observations and the theory on multipath propagation, the SNR values for both antennas were simulated following Equation (4) under the assumption of a horizontal reflector. Therefore, the multipath relative phase $\Delta \Phi_{M}$ was determined by Equation (6), and the attenuation factor α was derived from the raw SNR time series of G14 and G19 in units of dB-Hz, respectively, following the procedure described in Section 2.2. Finally, the trend of the simulated time series, which was modeled by a polynomial fit, was subtracted to determine the simulated δ-SNR time series. Since the spatial limitation of the reflector plane was not taken into account during the simulation, from a certain point onwards, the simulated and observed δ-SNR values were expected differ from each other. In Figure 20 and Figure 21, the observed and simulated δ-SNR time series of G14 and G19 are shown, respectively. For reasons of clarity, in both figures, the values are not color coded, but the percentage of overlap $P_{FZ}$ between the Fresnel zones and the roof is again grouped into the three phases: (1) $P_{FZ}=100\%$, (2) $50\%\leq P_{FZ}\geq 100\%$, and (3) $P_{FZ}\leq 50\%$.

For both satellites, the simulated and observed δ-SNR time series show very good agreement for $P_{FZ}=100\%$. This corresponds to the theoretical assumption that multipath will occur when the first Fresnel zones are completely located on the reflecting surface. The best agreement can be found for elevation angles lower than $30^{{}^{\circ}}$. In relation to the Rayleigh criterion and the smoothness of the reflecting surface, the assumption that the influence of diffuse reflections increases for elevation angles above $26^{{}^{\circ}}$ is confirmed. On the other hand, the simulated and observed SNR values differ greatly when the reflection point is not located on the roof and $P_{FZ}$ is smaller than 50%. Thus, the previous assumption that the variation in the δ-SNR time series during this phase shows random observation noise holds true. Furthermore, this corresponds to the propagation theory described in Section 2.1. Although Fresnel zones are not considered here, it is assumed that the reflection point is always on the reflector plane if multipath occurs. However, since the overlap between the Fresnel zones and the roof is always less than 50%, the Fresnel theory from Section 2.3 is also confirmed.

In contrast to the results of the comparison for $P_{FZ}=100\%$ and $P_{FZ}\leq 50\%$, the comparison of the simulated and observed δ-SNR values for the transition phase show partly ambiguous results. In the rising phase of G19 (cf. Figure 21), at both antennas, a good agreement between simulation and observation can be found. The same holds for G14 during the rising phase at antenna B and the setting phase at antenna A (cf. Figure 20). In the remaining transition phases, the agreement is less distinct. Nevertheless, the comparison reveals that especially during the transition phases with lower elevation angles, multipath occurs. Hence, it can be concluded that a Fresnel zone that is completely located on the reflecting surface does not necessarily need to be fulfilled as a theoretical prerequisite for multipath. In fact, the percentage of overlap between the Fresnel zone and reflector seems to be more important.

**Figure 22:**
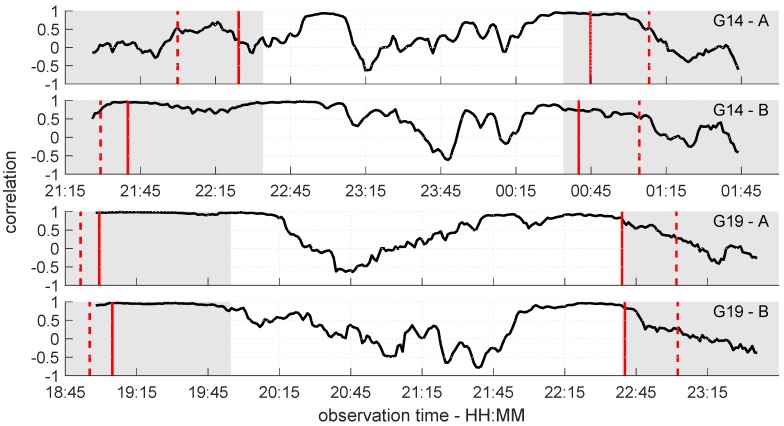
Correlation of simulated and observed δ-SNR time series for satellites G14 (**top** two panels) and G19 (**bottom** two panels). The grey shaded region represents the part of the observation duration where the Rayleigh criterion is fulfilled.

In order to numerically analyze the accordance of the simulated and observed δ-SNR time series, the time-dependent correlation $C_{t}$ was determined for a sliding window of 15 min length. The results are shown in Figure 22. Furthermore, the observation periods where the Rayleigh criterion was fulfilled are highlighted in grey, and the borders of the three phases of $P_{FZ}$ are denoted by the dashed and solid red lines.

Except for the black curve during the rising phase of G14 at antenna A (cf. top panel of Figure 22), the correlation of the simulated and observed δ-SNR time series corresponds to the visual analysis of Figure 20 and Figure 21. The highest correlations can be found when the Fresnel zone is completely located on the roof. From the time from where percentage of overlap decreases (solid red line), the correlation also decreases. Especially in the setting phases of both satellites, this becomes visible. Furthermore, the high variation of $C_{t}$ in the middle of the time series is reasonable, since in these periods, the Rayleigh criterion is not fulfilled. This leads to a higher probability of diffuse reflections, which are not modeled by the simulation. Nevertheless, since the correlation does not immediately drop at the borders between the grey and white areas, it is confirmed that the Rayleigh criterion cannot be interpreted as a sharp border between diffuse and specular reflection. The numerical analysis of the simulated and observed δ-SNR time series substantiates the findings from the visual analysis. The percentage of overlap between the Fresnel zone and the reflecting surface is the crucial factor for multipath occurrence.

Although the correlations show that the proposed approach is well suited for the analysis of the relationship between Fresnel zones and multipath occurrence, at the same time, limitations of this method are revealed. The simulation of the δ-SNR time series heavily relies on the assumption of a horizontal and smooth reflecting surface. If these assumptions are violated, the observed δ-SNR time series will differ from the simulations. For example a slight phase shift between both time series, as appears for antenna A during the rising phase of G14 (22:25 to 22:55), will directly lead to high differences and a decreasing correlation. Furthermore, the observed SNR-values can be influenced by effects, such as polarization loss or mismatch [36]. Although these effects are included in the attenuation factor α used for the simulation of the δ-SNR time series, they can also affect the correlation between observation and simulation.

## 5. Conclusions and Outlook

In the context of GPS, Fresnel ellipsoids describe the region between the satellite and user antenna where most of the signal energy is transmitted. The intersection of the ellipsoid with a reflecting surface leads to Fresnel zones, the active scattering regions that cause signal reflection and, in turn, lead to multipath effects after the superimposition with the signal received on the direct signal path.

Within this study, two aspects of using Fresnel zones in the context of GPS multipath were addressed. First, an algorithm for the identification of diffracted signals was proposed. Herein, Fresnel zones are determined for the line-of-sight transmission between the satellite and the user antenna. By comparing the borders of the Fresnel zone to an obstruction adaptive elevation mask, satellite signals that are potentially distorted by signal diffraction are identified and excluded from the position estimation process. The data of a field test was used to evaluate the proposed algorithm and it was found that both the positional accuracy and the percentage of epochs with fixed ambiguities improved.

Second, theoretical prerequisites for the occurance of multipath that are related to the location and size of the Fresnel zones were assessed. Therefore, the simulated and observed SNR time series of two antennas on a building roof were compared and related to the size and location of the Fresnel zones with respect to the spatially limited horizontal reflector. The results reveal that in contrast to the theoretical assumptions, multipath already can occur if the percentage of overlap between a Fresnel zone and a reflecting surface is above 50% and the reflection point of the satellite signal is located on the reflecting surface.

Although in most GNSS applications spatially limited reflectors are most often found in terms of buildings or other man-made objects, similar investigations in such environments are hard to realize. In particular, the separation of multipath effects induced from several reflectors is a non-trivial problem. Nevertheless, since Fresnel zones can also be determined on arbitrarily oriented reflectors, the findings of this study are transferable to more complex environments. In particular, the quality assessment of planned or already existing GNSS stations could profit from the enhanced detection of possible reflecting surfaces. This is subject to current research and is part of the further investigations in this context.

## Figures and Tables

**Table 1:** Surface roughness for different satellite elevation angles computed from the Rayleigh criterion for GPS-L1 carrier wavelength (λ=0.19 m).

**Elevation [°]**	5	10	20	30	40	50	60	70	80	90
**Δ*H* [cm]**	27.3	13.7	7.0	4.8	3.7	3.1	2.8	2.5	2.4	2.4

**Table 2:** (**a**) Minimum and maximum coordinate differences for the East-, North- and Up-component time series and the respective root-mean-squared error (RMS). (**b**) Percentage of outliers with respect to the 3σ boundary. (**c**) Percentage of observation epochs with fixed carrier phase ambiguities.

		Original Data Set			Classical OAEM			OAEM with Fresnel Zones		
		**Δ*E* [m]**	**Δ*N* [m]**	**Δ*U* [m]**	**Δ*E* [m]**	**Δ*N* [m]**	**Δ*U* [m]**	**Δ*E* [m]**	**Δ*N* [m]**	**Δ*U* [m]**
**(a)**	**min**	−0.158	−0.197	−0.361	−0.056	−0.018	−0.255	−0.013	−0.019	−0.050
	**max**	0.09	0.264	0.949	0.111	0.089	0.471	0.029	0.032	0.030
	**RMS**	0.003	0.004	0.008	0.003	0.004	0.008	0.003	0.004	0.007
**(b)**		**Percentage of outliers** (total number of epochs: 17,128)								
		1.0%	0.9%	1.2%	0.9%	0.8%	1.0%	0.6%	0.6%	0.5%
**(c)**		**Percentage of epochs with fixed ambiguities** (total number of epochs: 17,128)								
		95.5%			97.2%			99.9%		

## Abbreviations

The following abbreviations are used in this manuscript:

GNSS	Global Navigation Satellite System
GNSS-R	GNSS reflectometry
NLOS	Non-line-of-sight
OAEM	Obstruction adaptive elevation mask
RMS	Root-mean-square
RTK	Real Time Kinematic
SNR	Signal-to-Noise ratio
TLS	Terrestrial Laser Scanner
UAV	Unmanned Aerial Vehicle
VRS	Virtual Reference Station
